# Microcrystalline Hybridization Enhanced Coal‐Based Carbon Anode for Advanced Sodium‐Ion Batteries

**DOI:** 10.1002/advs.202200023

**Published:** 2022-05-04

**Authors:** He Chen, Ning Sun, Qizhen Zhu, Razium Ali Soomro, Bin Xu

**Affiliations:** ^1^ State Key Laboratory of Organic‐Inorganic Composites Beijing Key Laboratory of Electrochemical Process and Technology for Materials Beijing University of Chemical Technology Beijing 100029 China

**Keywords:** anodes, carbon materials, coal, heterostructures, sodium‐ion batteries

## Abstract

Sodium‐ion batteries (SIBs) are regarded as a kind of promising candidate for large‐scale energy storage technology. The development of advanced carbon anodes with high Na‐storage capacity and initial Coulombic efficiency (ICE) from low cost, resources abundant precursors is critical for SIBs. Here, a carbon microcrystalline hybridization route to synthesize hard carbons with extensive pseudo‐graphitic regions from lignite coal with the assistance of sucrose is proposed. Employing the cross‐linked interaction between sucrose and lignite coal to generate carbon‐based hybrid microcrystalline states, the obtained hard carbons possess pseudo‐graphitic dominant phases with large interlayer spaces that facilitate Na ion's storage and transportation, as well as fewer surface defects that guarantee high ICE. The LCS‐73 with an optimum cross‐link demonstrates the highest Na‐storage capacity of 356 mAh g^−1^ and an ICE of 82.9%. The corresponding full‐cell delivers a high energy density of 240 Wh kg^−1^ (based on the mass of anode and cathode materials) and excellent rate capability of 106 mAh g^−1^ at 10 C in addition to outstanding cycle performance with 80% retention over 500 cycles at 2 C. The proposed work offers an efficient route to develop high‐performance, low‐cost carbon‐based anode materials with potential application for advanced SIBs.

## Graphical abstract

A novel carbon microcrystalline hybridization route is proposed to synthesize hard carbon with enhanced sodium storage capacity and initial Coulombic efficiency (ICE). By the cross‐linking polymerization between sucrose and lignite coal, the obtained hard carbons possess extensive pseudo‐graphitic regions which facilitate Na‐ion storage and transportation, as well as fewer surface defects that guarantee high ICE.

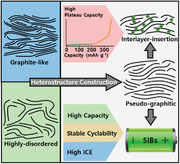

## 1 Introduction

The rapid development of large‐scale energy storage systems which are capable of storing renewable energy such as solar, wind, and hydro as electricity could lead to an established system that offers continuous energy supply to future energy storage devices.^[^ ^1^ ^]^ Lithium‐ion batteries (LIBs) with high energy density is a widely accepted power source for portable electronic devices and electrical vehicles.^[^ ^2^, ^3^, ^4^, ^5^ ^]^ However, the limited supplies of lithium restrict its potential uses, particularly in large‐scale energy storage systems. In contrast, sodium‐ion batteries (SIBs) are considered as a promising candidate for grid energy storage based on their satisfactory electrochemical performance, large reserves, and low cost.^[^ ^5^, ^6^, ^7^, ^8^ ^]^ Nevertheless, compared with Li ion (0.76 Å), the much larger ion radius of Na ion (1.02 Å) makes it difficult to find appropriate electrode materials for SIBs.^[^ ^9^, ^10^ ^]^ For example, graphite, a commercially successful LIBs anode, has poor Na‐storage performance with a reversible capacity of 35 mAh g^−1^ due to difficult intercalation of larger Na^+^ between the graphite interlayer as well as the graphite intercalation compound of Na is thermo‐dynamically more unstable than those of other alkalis.^[^ ^11^ ^]^ In this regard, various anode materials have been explored, such as carbonaceous materials,^[^ ^12^, ^13^, ^14^, ^15^, ^16^ ^]^ alloy,^[^ ^17^, ^18^, ^19^ ^]^ metal oxide/sulfide,^[^ ^20^, ^21^ ^]^ phosphorus,^[^ ^22^, ^23^ ^]^ and MXenes.^[^ ^24^, ^25^, ^26^, ^27^, ^28^, ^29^, ^30^ ^]^ The carbonaceous materials are relatively promising due to their low cost, abundant resource, environmental friendliness, and nontoxicity.^[^ ^14^, ^31^ ^]^ More importantly, amorphous carbons, especially hard carbons with highly disordered structures, could easily accommodate Na ion insertion/extraction allowing higher Na‐storage capacity.^[^ ^32^, ^33^, ^34^ ^]^

Hard carbon anode materials made from various precursors, including sucrose, glucose, and natural biomass, have been reported with high capacity (250–300 mAh g^−1^).^[^ ^35^, ^36^, ^37^, ^38^, ^39^ ^]^ However, the high irreversible consumption of Na ions at their abundant defect sites and the formation of solid electrolyte interface (SEI) during the first charge/discharge process often result in a poor initial coulombic efficiency (ICE).^[^ ^40^ ^]^ Furthermore, the low carbon output of these precursors is another impediment to the practical application of hard carbons.^[^ ^41^ ^]^ Thus, it is critical to investigate low‐cost precursors and design carbon anode materials with both high Na‐storage capacity and high ICE to realize their commercial applications.

Coal is a naturally occurring precursor to carbon with abundant reserves, low cost, and geographic distribution.^[^ ^42^, ^43^ ^]^ In addition, coal can be further categorized as anthracite, bituminous coal, and lignite coal based on its physicochemical characteristics, which could play a critical role in controlling the microcrystalline states of the final carbon materials. The relative cost‐benefit and structural diversity regard coal as a suitable platform to engineer efficient carbon materials for SIBs anode. Hu et al. prepared the carbon anode material for SIBs via direct pyrolysis of anthracite at 1200 °C, which exhibited a reversible Na‐storage capacity of 222 mAh g^−1^ owing to their ordered microstructure and smaller interlayer spaces.^[^ ^44^ ^]^ Similarly, Cao et al. prepared the carbon from sub‐bituminous coal through a simple one‐step carbonization process at 1300 °C, which delivered a much enhanced Na‐storage capacity of 291 mAh g^−1^ at a current density of 20 mA g^−1^.^[^ ^45^ ^]^ Nevertheless, its rate performance was still unsatisfactory, and a low capacity of only 57 mAh g^−1^ can be maintained at 400 mA g^−1^.

To date, various strategies have been considered to improve the electrochemical characteristics of these coal‐derived carbon‐based anodes, such as defect engineering, integrating coal‐based carbon bars,^[^ ^46^ ^]^ spheres,^[^ ^47^ ^]^ porous nanosheets,^[^ ^48^ ^]^ and composite formation based on the coal‐based graphite microcrystals.^[^ ^49^ ^]^ However, despite the high specific surface area and surface defects contribute to high sodium storage capacity, these carbon materials show poor ICE values (<60%). Recently, an extended “adsorption–insertion” model has been suggested that defines the role of the pseudo‐graphitic phase in hard carbon to enhance the plateau capacity efficiently.^[^ ^50^ ^]^ Thus, adjusting the carbon microcrystalline structure to a more pseudo‐graphitic phase, coal‐based hard carbon material with high capacity and ICE values could be anticipated.

Herein, we propose a carbon microcrystalline hybridization strategy to construct a coal‐based carbon material with the assistance of sucrose, which shows both high Na‐storage capacity and high ICE. The two precursors, lignite coal and sucrose, were cross‐linked polymerized in the carbonization process, leading to an improved structural stability of the precursor molecule. This microcrystalline hybridization method enabled carbon to show significantly enhanced pseudo‐graphitic phase with increased interlayer distance, allowing efficient Na‐ion insertion and transportation. In addition, the high structural stability of the cross‐linked molecules contributes to fewer carbon surface defects, allowing a high ICE value. The obtained carbon material with a larger pseudo‐graphitic content and less surface defects achieved a high Na‐storage capacity of 356 mAh g^−1^ with an ICE value of 82.9%, as well as excellent cycle performance and rate capabilities, indicating a promising anode material for advanced SIBs.

## 2 Results and Discussion

**Figure 1:**
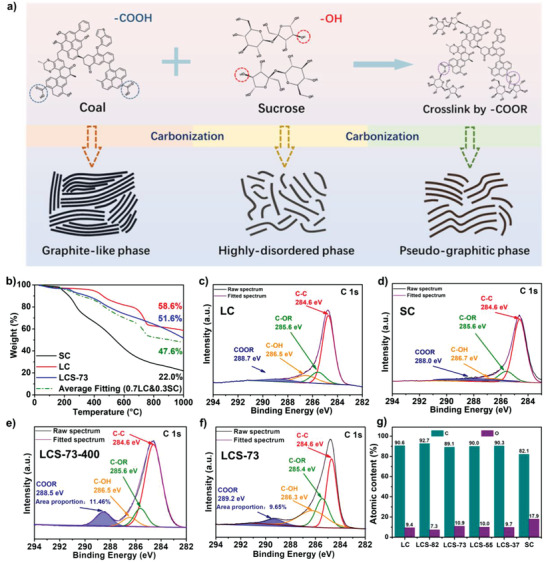
a) Schematic diagram for the fabrication of microcrystalline hybrid coal‐based carbon and the characteristics of the microcrystalline structure. b) TGA curve of lignite coal, sucrose, and the mixture of lignite coal and sucrose. High‐resolution C 1s spectra of c) LC, d) SC, e) LCS‐73‐400, and f) LCS‐73. g) Corresponding element content of all the samples estimated using XPS survey spectra.

Due to the different molecular compositions of precursors, the lignite coal‐pyrolyzed carbon (LC) shows graphite‐like phase dominant carbon microcrystalline structure with smaller interlayer spaces, while the sucrose‐derived carbon (SC) mainly consists of highly disordered carbon phase. Both of pure lignite coal‐pyrolyzed carbon and sucrose‐pyrolyzed carbon are not attractive for sodium‐ion storage. With the assistance of sucrose, a microcrystalline hybridized lignite coal‐based carbon (LCS) with much pseudo‐graphitic phase was prepared, as shown in **Figure** **1**a. Initially, lignite coal powder was mixed with sucrose in a certain ratio and the mixture was compressed to facilitate the close contact of the two components followed by thermal treatment at 400 °C. The Fourier transform infrared spectroscopy (Figure S1a, Supporting Information) confirms the cross‐linking polymerization between the sucrose and the lignite coal. It should be noted that LCS contains C═O (COOR) framework vibration at 3436 cm^−1^ and C—O (COOR) framework vibration at 1386 cm^−1^, which are attributed to the cross‐linking reaction between ‐OH groups of the sucrose and the ‐COOH groups of the lignite coal. Obviously, LCS‐73 exhibits much sharper peaks than other samples, indicating a more sufficient cross‐linking reaction. The presence of sucrose molecules could effectively inhibit the microcrystalline ordering degree of the coal‐derived carbon, resulting in the increased pseudo‐graphitic phase with large interlayer spacing during the pyrolysis at 1200 °C.^[^ ^50^ ^]^ To ensure optimum structural composition, carbon material was prepared using different lignite coal/sucrose mass ratios of 8:2, 7:3, 5:5, and 3:7, denoted as LCS‐82, LCS‐73, LCS‐55, and LCS‐37, respectively. Pristine carbon materials were prepared from pristine lignite coal and sucrose for analytical comparison using similar experimental conditions. Thermogravimetric analysis (TGA) was carried out under Ar atmosphere from room temperature to 1000 °C at 5 °C min^−1^ to study the pyrolysis behavior of different precursors (Figure 1b and Figure S1b, Supporting Information). As shown in Figure 1b, lignite coal, sucrose, and the lignite coal/sucrose mixture (7:3) exhibited an obvious weight loss of 41.4%, 78.0%, and 48.4% as the temperature rose from room temperature to 400 °C, which could be attributed to the loss of both oxygen and hydrogen. The relatively rapid mass loss detected at 400–500 °C indicates mainly the production of gas, tar, and other volatile matter.^[^ ^51^ ^]^ Unlike pristine lignite coal, which showed a rapid mass loss of 14.5% between 700 and 760 °C due to the rapid decomposition of aliphatic hydrocarbon and hydrogenated aromatic rings forming aromatic components as volatile matter,^[^ ^52^, ^53^, ^54^ ^]^ the lignite coal/sucrose mixture realized a smooth mass loss due to enhanced molecular structural stability (Figure S1b, Supporting Information). In addition, the TGA curve of LCS‐73 is distinct from the fitting curve calculated based on the mass ratio of LC and SC (7:3), indicating the changed pyrolysis behavior due to the cross‐linking reaction between the lignite coal and the sucrose molecules. The obtained carbon yield of 51.6% for LCS‐73 (blue line) at the pyrolysis temperature of 1000 °C is higher than the calculated value of 47.6% (green line). Moreover, as shown in Table S1 in the Supporting Information, the experimental carbon yields of the hybrid carbons pyrolyzed at 1200 °C are all higher than the theoretical values calculated from the carbonation yields of pure lignite coal and sucrose. These results confirm the successful cross‐linking interaction between lignite coal and sucrose, which greatly enhances the structural stability of the precursors.

To further reveal the interaction mechanism in the cross‐linked lignite coal and sucrose, X‐ray photoelectron spectroscopy (XPS) analysis was carried for the precursors of lignite coal and sucrose, the intermediate products after thermal treatment at 400 °C (LC‐400, SC‐400, and LCS‐73‐400), as well as the obtained LCS samples with various lignite coal/sucrose mass ratios. The most obvious peaks at 284.6 and 533.3 eV in the survey spectra (Figure S2, Supporting Information) are designated as C 1s and O 1s, respectively, with the O 1s intensity increasing from LCS‐82 to LCS‐37. The C 1s (Figure 1c–f) was deconvoluted into C—C (284.6 eV), C—OH (286.5 eV), —COOR (288.7 eV), and C—OR (285.2 eV). The O 1s (Figure S3, Supporting Information) was resolved into O‐I (‐COOR, 533.4 eV), O‐II (phenol groups or ether groups, 532.3 eV), and O‐III (carbonyl groups, 531.4 eV).^[^ ^12^, ^55^, ^56^ ^]^ Unlike LC and SC (Figure 1c,d) which have minimal presence of ‐COOR groups, the LCS‐73 shows higher proportion of ‐COOR groups as evident from the C 1s (Figure 1f) and O 1s (Figure S3, Supporting Information) profiles. The C 1s (Figure 1e) and O 1s (Figure S4, Supporting Information) profiles for the intermediate precursor LCS‐73‐400 also indicate high proportion of ‐COOR groups, implying the occurrence of cross‐linking to form ester bonds between lignite coal and sucrose. Compared with other LCS samples (LCS‐82, LCS‐55, and LCS‐37) as exhibited in Figure S5 in the Supporting Information, LCS‐73 presents the highest proportion (9.65%) of ‐COOR groups in the C 1s and also the highest proportion (66.39%) of O‐I in O 1s profiles (Table S2, Supporting Information), indicating the most sufficient cross‐linking reaction of LCS‐73. Besides, the LCS samples possess high carbon content of ≈90 at% (Figure 1g) meaning the complete carbonization. Element analysis (Table S3, Supporting Information) indicates the carbon content of the hybrid carbons LCS‐73 and LCS‐82 are 93.93 and 94.80 wt%, respectively, which are larger than those of the pure lignite coal‐derived carbon (93.01 wt%) and sucrose‐derived carbon (87.94 wt%), confirming its enhanced structure stability of the hybrid carbon precursor due to the efficient cross‐linking reaction between the lignite coal and the sucrose molecules.^[^ ^57^ ^]^ The above results confirm the stable cross‐linking in the LCS‐73 even when carbonization temperature increases to 1200 °C with a microcrystalline hybridized structure rich in pseudo‐graphic regions.

**Figure 2:**
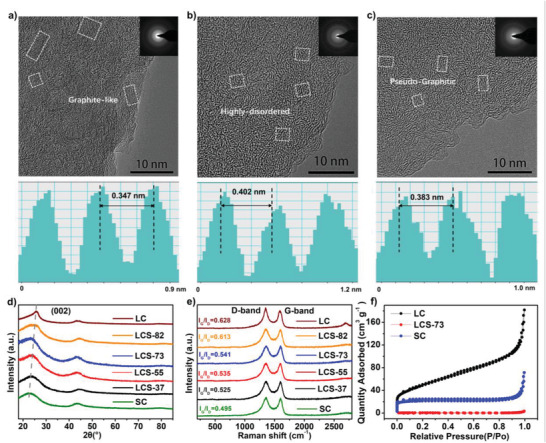
HRTEM images and the corresponding FFT patterns of the carbon materials: a) LC, b) SC, and c) LCS‐73. d) XRD patterns, e) Raman spectra and f) nitrogen adsorption/desorption isotherms of LC, SC, and LCS samples.

In order to confirm the carbon microcrystalline hybridization structure of the LCS samples, the high‐resolution transmission electron microscope (HRTEM) images of LC, SC, and LCS‐73 carbons with the corresponding selected area electron diffraction (SAED) are displayed in **Figure** **2**a–c, and the scanning electron microscope (SEM) images of LCS‐73 show an irregular particle morphology, as displayed in Figure S6 in the Supporting Information. For the LC, plenty of graphite‐like phase with the clearly visible long‐range‐ordered crystallite stripes can be observed (Figure 2a), while the SC exhibits highly disordered microstructures (Figure 2b). It is noteworthy that the LCS‐73 shows a typical pseudo‐graphitic structure based on the carbon microcrystalline hybridization rather than a simple mixture of LC and SC (Figure 2c). The corresponding SAED patterns also follow a similar trend with diffraction rings gradual diffusion as structural changes from graphite‐like to pseudo‐graphitic phase. The fast Fourier transform (FFT) pattern measured the average fringe spacing of 0.347, 0.402, and 0.383 nm for LC, SC, and LCS‐73, respectively, indicating the existence of a relatively higher pseudo‐graphitic phase (d‐spacing in 0.36–0.40 nm) in LCS‐73. The carbon in the pseudo‐graphitic phase could easily be accessed by Na ion, realizing a high theoretical capacity of 279 mAh g^−1^ in the low‐potential (<0.1 V) plateau region based on the “interlayer‐insertion” mechanism.^[^ ^50^ ^]^ Thus, the LCS‐73 with much pseudo‐graphitic phase could be anticipated as high‐performance anode material for SIBs.

The X‐ray diffraction (XRD) and Raman spectra results further confirmed the microstructures of the carbon samples. Figure 2d shows the XRD patterns with two obvious broad peaks at 23° and 44°, corresponding to the typical diffraction of (002) and (100) planes, respectively. The LC displays a prominent peak at 25.7°, indicating a d‐spacing of 0.345 nm and a rather ordered carbon microcrystallites, whereas SC has a broad (002) peak at 22.7°, reflecting disordered microstructure and d‐spacing up to 0.396 nm. In the case of LCS, the typical (002) peak falls between that of LC and SC, indicating an intermediated spacing of 0.36–0.39 nm. Furthermore, when the content of sucrose in the precursor increases from 20% to 70%, the (002) peak shifts to lower angles, suggesting a more amorphous carbon microstructure with larger interlayer spaces. Raman spectral analysis confirms the presence of two characteristic bands at ≈1345 cm^−1^ (D‐band) and ≈1600 cm^−1^ (G‐band), representing disordered and graphitic structures. The intensity ratio of the G‐band to the D‐band (*I*_G_/*I*_D_) can be used to demonstrate the structure ordering of the carbon microcrystalline.^[^ ^58^ ^]^ The *I*_G_/*I*_D_ of LC was determined to be 0.628, indicating a relative high graphitization degree, while that of SC was calculated to be 0.495 corresponding to a highly disordered structure. The *I*_G_/*I*_D_ values for LCS were in the range of 0.613–0.525, which agreed with the XRD analysis.

In general, the hard carbon microstructures are inhomogenous with the coexistence of distinct microcrystallites, as shown by HRTEM images and confirmed using XRD investigation. According to the literature, the (002) peaks in the XRD pattern could be simulated to analyze the evolution of the carbon microcrystalline state against increasing sucrose concentration (Figure S7, Supporting Information and **Table** **1**).^[^ ^58^ ^]^ In this case, LC carbon with a high graphitization degree possesses a high proportion of graphite‐like phases with a d‐spacing below 0.36 nm, accounting for 64.66% in the microcrystalline (Figure S7a, Supporting Information). The addition of sucrose can inhibit the formation and growth of graphite microcrystalline, and as the sucrose concentration increases, graphite‐like phases gradually transform to pseudo‐graphitic phases, reaching a maximum value of 71.38% in LCS‐73. As the sucrose concentration rises in LCS‐55 and LCS‐37, a highly disordered phase gradually appears and grows in proportion to the sucrose content resulting in the declined pseudo‐graphitic region from 69.17% to 43.24%. This result indicates an optimum addition content of sucrose is important for sufficient cross‐linking to obtain pseudo‐graphitic phase‐predominant carbon anode, leading to the optimized sodium storage performances in SIBs, from the view of capacity, ICE, and cycle stability.

**Table 1:** The XRD (002) peak fitting analysis for LCS samples

	Highly disordered				Pseudo‐graphitic				Graphite‐like		
	2*θ* [°]	*d*_002_ [nm]	Area [%]		2*θ* [°]	*d*_002_ [nm]	Area [%]		2*θ* [°]	*d*_002_ [nm]	Area [%]
LC	–	–	–		23.02	0.386	35.34		25.81	0.345	64.66
LCS‐82	–	–	–		23.08	0.385	62.70		26.05	0.342	37.30
LCS‐73	–	–	–		23.43	0.379	71.38		26.24	0.339	28.62
LCS‐55	21.46	0.414	30.83		24.55	0.362	69.17		–	–	–
LCS‐37	21.42	0.414	56.76		24.71	0.360	43.24		–	–	–
SC	21.89	0.406	70.65		24.54	0.362	29.35		–	–	–

Aside from the appropriate interlayer spacing for Na‐ion insertion/extraction, minimal surface defects are another key parameter for carbon anode materials in SIBs since the former offers high Na‐storage capacity and the latter assures high ICE. The N_2_ (77 K) adsorption/desorption measurements were used to study the porosity of the carbon samples (Figure 2f and Figure S8, Supporting Information), while Table S4 in the Supporting Information shows the calculated Brunauer–Emmett–Teller (BET) surface area and pore volumes of the samples. As seen, the LC and SC have relatively greater surface defects with a surface area of 169.52 and 80.69 m^2^ g^−1^, respectively. However, LCS‐73 realizes a BET surface area of only 1.48 m^2^ g^−1^, suggesting the negligible porosity. In this case, the cross‐linked molecular structure of the LCS‐400 precursor inhibits the release of gas small molecules during high‐temperature treatment, thus significantly reducing surface defects formation. These minimal surface defects are critical to avoiding large irreversible capacity and realizing a high ICE value during the initial charge/discharge process.

**Figure 3:**
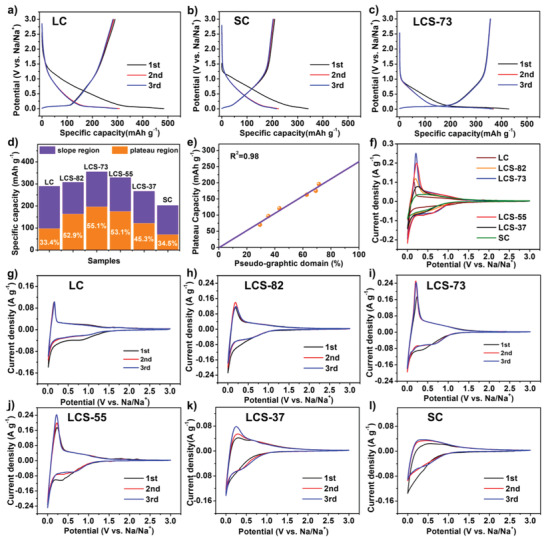
Electrochemical Na‐storage behavior of the prepared carbon samples. Galvanostatic charge/discharge profiles in the initial three cycles at 0.03 A g^−1^ of a) LC, b) SC, and c) LCS‐73. d) The plateau region capacity and sloping region capacity comparison in various carbon samples (based on the third charge curve). e) The plateau region capacity as a function of the carbon crystallite's pseudo‐graphitic domain. f) CV curves for all samples at a scan rate of 0.1 mV s^−1^, and first three CV cycles of g) LC, h) LCS‐82, i) LCS‐73, j) LCS‐55, k) LCS‐37, and l) SC at 0.1 mV s^−1^.

The electrochemical Na‐storage behaviors of the prepared carbons were studied using galvanostatic charge/discharge profiles in initial three cycles (**Figure** **3**a–c). The test was performed using a half‐coin cell and Na foil as the counter electrodes with 1 m NaClO_4_ (ethylene (EC) + diethyl carbonate (DEC), 5% fluoroethylene carbonate (FEC)) as the electrolyte. The profiles of all the samples consist of two regions, i.e., the sloping region above 0.1 V and the plateau region below 0.1 V, corresponding to the surface adsorption and interlayer insertion mechanism for Na‐ion storage, respectively.^[^ ^50^ ^]^ As shown in Figure 3a, LC exhibits a reversible Na‐storage capacity of 290 mAh g^−1^ with an ICE of only 59.9% due to the relative ordered microcrystalline structure and ample surface defects. In addition, as its microcrystalline is mostly composed of the graphite‐like (interlayer distance below 0.36 nm) domains with the ratio of 64.7%, unfavorable for Na ion insertion/extraction, the LC presents a small plateau region capacity of 110 mAh g^−1^. On the other side, SC exhibits a low capacity of 209 mAh g^−1^ with the sloping region dominating the charge/discharge curves based on their highly disordered microcrystalline structure with d‐spacing larger than 0.40 nm, which enables Na ion to be stored in the layers by adsorption mechanism.^[^ ^59^ ^]^ In contrast, the LCS‐73 displayed a much enhanced reversible capacity of 356 mAh g^−1^ with a dominance of low‐voltage plateau region for total Na‐storage capacity based on relatively greater pseudo‐graphitic phases consisting of suitable interlayer spacing. Moreover, the minimal surface defects further improve the ICE value of LCS‐73 from 59.9% of LC to 82.9%, which is appealing for commercial applications.

Since the sucrose to coal ratio has a considerable influence on the cross‐linking degree and the microstructures of the carbonized products, the LCS carbons prepared with different coal/sucrose ratios show different capacities and ICE values. Figure S9 in the Supporting Information shows that the LCS‐82, with 20% sucrose, has an increased Na‐storage capacity of 317 mAh g^−1^ and a higher ICE of 76.4% compared to its LC counterpart. The degree of cross‐linking between the coal and the sucrose increases as sucrose content increases, and the optimal reaction was reached at an LCS set ratio of 7:3 (LCS‐73) which achieved a high Na‐storage capacity of 356 mAh g^−1^ and a high ICE of 82.9%. The sucrose has an abundance of oxygen‐containing functional groups compared to coal, and thus a stronger cross‐linked reactivity was achieved at the optimum sucrose to coal ratio of 7:3. As the sucrose concentration is further increased, the resulting carbons, LCS‐55 and LCS‐37, realize decreased Na‐storage performance than LCS‐73. The charge/discharge curves of the LCS carbons could further be divided into a plateau region below 0.1 V, and a sloping region above 0.1 V, indicating the co‐contribution of surface adsorption and interlayer insertion to the capacity.

Figure 3d shows the capacity distribution along the plateau and sloping region for LCS carbons where the proportion of plateau capacity increases from 33.4% for LC to 52.9% for LCS‐82. The introduction of sucrose causes carbon microcrystalline hybridization allowing interlayer expansion and promoting interlayer insertion‐based Na‐storage capacity. The plateau region ratio reaches the maximum value of 55.1% in LCS‐73, indicating that the cross‐linking reaction of the two precursors results in optimum carbon microcrystalline hybridized structure with much more pseudo‐graphitic phase and suitable interlayer structure (0.36–0.40 nm) for Na‐ion insertion/extraction. In the case of LCS‐55 and LCS‐37, the plateau capacity declines, indicating that the excessive sucrose could cause an insufficient cross‐linking reaction and formation of undesirable surface defects, leading to a capacitive‐adsorption contribution for Na‐ion storage that corresponds to sloping region in the charge/discharge curve.

Figure 3e shows a linear correlation between plateau capacity and pseudo‐graphitic phase with a correlation coefficient of 0.98. The fitting curve passes through zero point and follows the relationship defined as *Y* = 266(*X*). It means that if the pseudo‐graphitic domain reaches 100%, a plateau capacity of 266 mAh g^−1^ will be obtained, which is near the predicted maximum plateau capacity of 279 mAh g^−1^ for NaC_8_ formation, correlated well with previous report.^[^ ^50^ ^]^

Cyclic voltammetry (CV) curves were also performed to analyze the electrochemical behavior of the carbon samples (Figure 3f–l) where an irreversible reduction broad peak around 0.2–0.8 V could be seen during the initial cycles corresponding to the formation of SEI film. The sharp peak at ≈0.1 V with a broad hump at 0.2–2 V corresponds to the plateau and sloping region of the charge/discharge curves, respectively. The intensity variation of the sharp peak at 0.1 V coincides well with the plateau region capacity in the charge/discharge curves for carbons with various coal/sucrose ratios. For example, the LC has a weaker peak at ≈0.1 V compared to LCS, and LCS‐73 exhibits the sharpest peak at ≈0.1 V corresponding to the largest plateau region capacity in charge/discharge profile. As the sucrose content in the precursor further increases, the peak at 0.1 V gradually weakens from LCS‐73 to LCS‐37 and transforms into a broad hump in the case of SC, consistent with the plateau region capacity variation.

**Figure 4:**
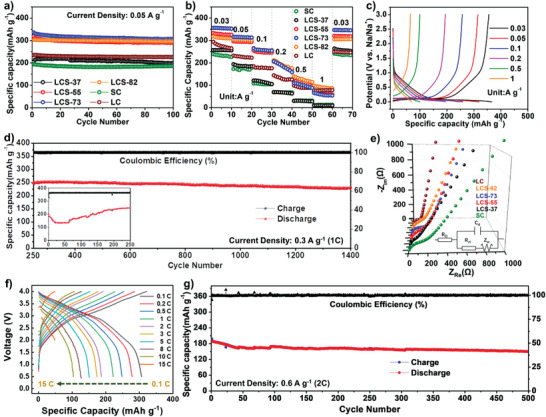
Electrochemical Na‐storage performances of the carbon samples. a) Cycle performance at 0.05 A g^−1^; b) rate performance at different current densities; c) galvanostatic charge/discharge profiles of LCS‐73 at different current densities; d) cycle performance of LCS‐73 at 0.3 A g^−1^; e) EIS of the samples; f) galvanostatic charge/discharge profiles of the LCS‐73//O3‐NaNi_1/3_Fe_1/3_Mn_1/3_O_2_ full‐cell at different current densities; g) cycle performance of the LCS‐73//O3‐NaNi_1/3_Fe_1/3_Mn_1/3_O_2_ full‐cell at 2 C.

The cyclic stability was evaluated under a constant current of 0.05 A g^−1^ (**Figure** **4**a). In this case, LCS‐73 could retain a high capacity of 307 mAh g^−1^ over 100 cycles with a capacity retention of 91.3%, which implies the outstanding structural durability of the microcrystalline hybridization structure for the repeating Na^+^ insertion/extraction process. The LCS‐73 also exhibits good rate performance, as shown in Figure 4b. Due to the large pseudo‐graphitic phase and balanced plateau region capacity, the LCS‐73 could display the highest reversible capacity of 356, 316, 265, and 207 mAh g^−1^ at the current density of 0.03, 0.05, 0.1, and 0.2 A g^−1^ respectively, outperforming SC and LC. Since the rate capability depends on electrode materials' electronic and ionic conductivity, the LCS‐82 with a relatively more graphite‐like phase and superior electronic conductivity exhibits a better rate performance of 139 mAh g^−1^ at a larger current density of 0.5 A g^−1^. In comparison to the previously known coal‐based carbon anodes materials, such as pyrolyzed bituminous coal (65 mAh g^−1^ at 0.4 A g^−1^) and pyrolyzed anthracite (80 mAh g^−1^ at 0.6 A g^−1^), the superiority in electrochemical Na‐storage performance of the microcrystalline hybridized LCS carbons can also be highlighted (Figure S10, Supporting Information).^[^ ^44^, ^60^ ^]^

The galvanostatic charge/discharge profiles of LCS‐73 at different current densities were plotted to study the variation of electrochemical behavior with the current rate (Figure 4c). As the current density increases, the plateau region curve gradually diminishes and even disappears when the current density reaches 0.5 A g^−1^. The fast capacity decay in the plateau region is responsible for the overall capacity fading at such high rates due to the slower kinetics of plateau region associated with interlayer insertion than sloping region related to surface adsorption. Thus, the sloping and plateau regions must be balanced to realize the highest overall electrochemical performance.

The predominant surface adsorption mechanism for Na‐ion storage at higher current rates suggests that the capacitive adsorption is dynamically faster than the interlayer insertion mechanism. Figure 4d shows the long‐term cycle performance of LCS‐73 electrode at a high current density of 0.3 A g^−1^. In the initial cycles, the electrode material presents an activation process, after which the capacity stabilizes at 250 mAh g^−1^. Noteworthy, the capacity can still remain 225 mAh g^−1^ over 1400 cycles with a retention of 90.0%, verifying the excellent high‐rate cycle stability of the microcrystalline hybridized LCS carbons. Figure 4e shows the electrochemical impedance spectroscopy (EIS) of the fresh carbon electrodes measured at the charging state (≈2.6 V vs Na^+^/Na) with the corresponding equivalent circuit. In the EIS curves of all the samples, a semicircle and a sloping line could be distinguished, which is related to the resistance of the charge transfer (*R*_ct_) and ion diffusion process (*W*), respectively.^[^ ^61^, ^62^, ^63^ ^]^ The *R*_ct_ values increase from 94.1 Ω (LCS‐82) to 160.4 Ω (LCS‐37) with increasing sucrose content (Table S5, Supporting Information), which is in well agreement of the above rate performance. Moreover, the steep slopes of LC, LCS‐82, and LCS‐73 in the low‐frequency region indicate the fast ionic diffusion.

To further verify the application prospects of the as‐prepared coal‐based carbons with microcrystalline hybridization structure, coin‐type full cells were assembled using LCS‐73 as anode material, O3‐NaNi_1/3_Fe_1/3_Mn_1/3_O_2_ as cathode material, and 1 m NaClO_4_ (1:1 EC+DEC, 5% FEC) as electrolyte. The corresponding charge/discharge profiles (Figure 4f) were recorded in the voltage range of 0.5–4 V at various current rates (0.1 C–15 C, 1 C = 300 mA g^−1^). The full‐cell exhibited a high reversible capacity of 307 mAh g^−1^ at 0.1 C with an ICE of 95.4% based on the mass of anode material, and could remain 106 mAh g^−1^ at a high rate of 10 C. Moreover, the full‐cell realized a maximum practical energy density of 240 Wh kg^−1^ based on the total mass of the anode and cathode with a stable cycle performance. In the full‐cell, the capacity based on anode is 189 mAh g^−1^ at 2 C, with a capacity retention ratio of 83.3% after 500 cycles (Figure 4g). These results confirm that the sodium‐storage performance of the LCS‐73 is relatively superior to most of the hard carbon anodes reported previously,^[^ ^44^, ^64^, ^65^, ^66^, ^67^ ^]^ supporting its promising applications as advanced SIB anodes.

**Figure 5:**
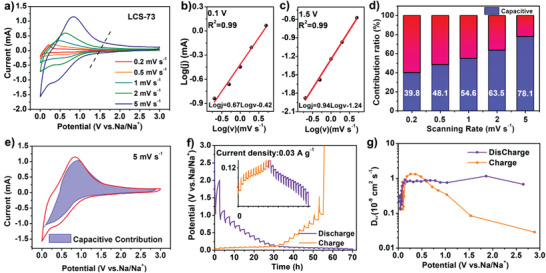
Electrochemical kinetics analysis for the microcrystalline hybridized LCS‐73. a) CV curves at various scan rates from 0.2 to 5 mV s^−1^. The correlations of current scan rate (*v*) and peak current (*i*) around b) 0.1 V and c) 1.5 V during the discharge process. d) Capacitive contributions at various scanning rates. e) CV curve with a calculated capacitive contribution at 5 mV s^−1^. f) GITT potential profiles and g) the calculated Na^+^ chemical diffusion coefficients during the charge/discharge process.

To investigate the charge storage mechanism in the microcrystalline hybridized carbon, kinetic analysis was carried based on the CV curves of LCS‐73 at various scanning rates (0.2–5 mV s^−1^) (**Figure** **5**a). The scan rate (*v*) and peak current (*i*) could be related using the following equation^[^ ^68^, ^69^ ^]^

(1) $$i\;=\;a\nu^{b}$$

where *a* and *b* are the constants, and *b* could be estimated from the slope curve plotted between the log(*v*) versus log(*i*). The *b* value of 0.5 indicates a diffusion‐controlled process, whereas the *b* value of 1 corresponds to the surface‐controlled reactions. Considering the potential deviation from equilibrium potential caused by irreversible resistance,^[^ ^68^ ^]^ we take points according to the potential deviation as shown in Figure 5a around 0.1 and 1.5 V. Figure 5b,c shows the plot for log(*v*)−log(*i*) around 0.1 and 1.5 V with a good linear relationship (*R* ^2^ = 0.99). As evident, the *b* value estimated from the slope of the curve at 0.1 V was 0.67, reflecting a diffusion‐dominant sodium‐storage process in the low‐potential region. In contrast, the *b* value of 0.94 was obtained for the curve plotted at 1.5 V representing surface‐controlled reactions such as surface adsorption in the high‐potential region. To quantitatively determine the contribution from the surface‐controlled process and diffusion‐controlled process, the CV curves of LCS‐73 were further analyzed using the following formula^[^ ^70^, ^71^ ^]^

(2) $$i\left(V\right)\;=k_{1}\;\nu +k_{2}\nu^{1/2}$$

here, *ν* stands for the scan rate, and *k*_1_ and *k*_2_ can be easily determined from the plot of *i*(*V*)*/ν* ^1/2^ versus *ν* ^1/2^ plots.^[^ ^39^ ^]^ The *k*_1_ *ν* and *k*_2_ *ν* ^1/2^ refer to the surface capacitive effect and diffusion‐controlled insertion process, respectively. Accordingly, 39.8% of the current resulted from the nondiffusion‐controlled process at the scan rate of 0.2 mV s^−1^. As the scan rate increases, the contribution from diffusion‐controlled process diminishes whereas the nondiffusion‐controlled contribution enhances (Figure 5d). The nondiffusion‐controlled process, i.e., capacitive contribution, reaches up to 78.1% at 5 mV s^−1^ (Figure 5e). The nondiffusion‐controlled process with faster kinetics dominates the charge transportation at high rates enabling excellent rate performance of the microcrystalline hybridized carbon materials, i.e., LCS‐73. To further verify the enhanced rate capability of LCS‐73 by kinetic analysis, the capacitive nature of LC was investigated as exhibited in Figure S11 in the Supporting Information. The LCS‐73 shows higher capacitive contribution than LC at different scanning rates, e.g., capacitive contribution of LCS‐73 is 78.1%, while LC possesses only 64.1% at 5 mV s^−1^, respectively. The higher capacitive nature of LCS‐73 results in enhanced rate capability and improved cyclability, and thus an overall improvement in the electrochemical performances.

The kinetics of Na‐ion transportation in LCS‐73 was determined using the galvanostatic intermittent titration technique (GITT). Figure 5f shows the GITT potential profiles of LCS‐73 with a pulse current of 0.03 A g^−1^. The corresponding diffusion coefficients of Na^+^ were calculated based on the following formula^[^ ^72^, ^73^ ^]^

(3) $$D\;=\frac{4}{\pi \tau }\;{\left(\frac{n_{m}V_{m}}{S}\right)}^{2}{\left(\frac{\Delta E_{s}}{\Delta E_{t}}\right)}^{2}$$

In this formula, *n*_m_, *V*_m_, *τ*, and *S* are the mole of the electrode material, molar volume, relaxation time, and electrode–electrolyte contact area, respectively. In addition, the enlarged GITT potential profiles are exhibited in Figure S12 in the Supporting Information, in which Δ*E*_s_ is the potential change caused by pulse and Δ*E*_t_ is the potential change for constant current charging (discharging). Figure 5g shows the apparent diffusion coefficients of Na^+^ (*D*_Na_ *^+^*) as a function of potential. During the sodiation process (discharging), the calculated *D*_Na_^+^ values maintain a high value in the high‐potential region which later decreases rapidly from 0.2 to 0.001 V, indicating that surface adsorption mechanism in the high‐potential sloping region has a faster dynamics than diffusion‐controlled process, i.e., interlayer insertion mechanism, in the low‐potential plateau region. Additionally, the *D*_Na_^+^ first increases from 0.001 to 0.25 V during the desodiation stage (charging) and then declines before reaching the cut‐off potential.^[^ ^74^ ^]^ This decrease from 0.25 to 3 V could be attributed to the reduction of Na^+^ concentration in the electrode material.^[^ ^75^ ^]^ The GITT analysis indicates that LCS‐73 exhibits much higher diffusivity coefficient value than LC and SC in the plateau region (Figure S13, Supporting Information).The difference in the diffusivity coefficient values in sloping and plateau regions indicated that there are different binding energies for the Na^+^ interactions.^[^ ^31^ ^]^ It should be noted that the LC and SC present the lower Na^+^ ions diffusion coefficients than LCS‐73, which sheds light on the good electrochemical performance of LCS‐73.

## 3 Conclusions

In summary, we propose an effective strategy to control the microcrystalline structure of coal‐based carbon material for enhanced sodium‐storage capacity with a high ICE value. The two precursors, lignite coal and sucrose were subjected to cross‐link interaction to form carbon‐based hybrid microcrystalline states, where interlaced sucrose molecules assisted in the carbonization process. The structural modification enabled the engineering of the pseudo‐graphitic phase with favorable interlayer spacing for enhanced Na^+^ insertion/extraction, as well as realized fewer surface defects due to stable structural configuration, resulting in relatively higher ICE values. The electrochemical characteristics were studied for hybrid microcrystallite with various sucrose‐to‐coal ratios, with LCS‐73 exhibiting the highest Na‐storage capacity of 356 mAh g^−1^ and an ICE value of 82.9% based on optimum pseudo‐graphitic regions and adequate conductivity. The full‐cell was assembled with LCS‐73 as anode delivered a high energy density of 240 Wh kg ^−1^ with outstanding rate capability (106 mAh g^−1^ at 10 C) besides excellent cycle stability with 80% retention over 500 cycles at 2 C. In general, the microcrystalline hybridization is a very simple but effective strategy to develop advanced carbon anode materials combining excellent Na‐storage performance and low cost for practical SIBs, which can also be extended to the microcrystalline structure adjustment of carbonaceous materials in other secondary ion batteries.

## 4 Experimental Section

### Material Synthesis

The microcrystalline hybridized carbon materials (LCS) were prepared from two precursors, lignite coal (Xin Jiang province, China) and sucrose (Tianjin Yongda Co., China, 99.6%). Typically, lignite coal and sucrose were mixed by the ball‐milling method in a certain ratio followed by compression to form tablets. The obtained tablets were heated at 400 °C for 2 h to induce a cross‐linking reaction of lignite coal and sucrose followed by thermal treatment at 1200 °C in a tube furnace for 2 h with the heating rate 5 °C min^−1^ under argon flow. The prepared carbons with the lignite coal/sucrose mass ratio of 3:7, 5:5, 7:3, 8:2 were donated as LCS‐37, LCS‐55, LCS‐73, LCS‐82, respectively. In comparison, carbon materials were also prepared via direct pyrolysis of pristine lignite coal and sucrose at 1200 °C, denoted as LC and SC, respectively.

### Materials Characterizations

The morphologies of the prepared carbon materials were determined using an SEM (Hitachi SU8000). The HRTEM images and SAED patterns were recorded by a field‐emission TEM (Tecnai G2 F30). The microcrystalline structures of carbon materials were characterized using an X‐ray diffractometer (Ultima IV, Japan) with Cu‐K*α* radiation (1.5405 Å), and Raman spectra using a confocal micro Raman spectrometer (Renishaw, inVia Reflex, England). The nitrogen adsorption–desorption isotherms were recorded using a Micromeritics ASAP2460 analyzer. The pore size distribution were calculated by a density functional theory method. XPS spectra were obtained using an X‐ray photoelectron spectrometer (ESCALAB 250, Thermo VG, American). The element analysis was tested in Elemental Analyzer (varioELcube, Germany). The TGA was carried out using a synchronous thermal analyzer (TGA/DSC3+, Mettler) with a temperature range of 40 to 1000 °C at 5 °C min^−1^ under Ar gas atmosphere.

### Electrochemical Measurements

All the electrochemical tests were performed in coin cells (C2025‐type). The working electrodes were prepared by spreading the uniform slurry of active material and carboxymethylcellulose binder in water solvent with a mass ratio of 95:5 onto the Cu foil current collector. After being dried under vacuum at 120 °C for 12 h, the electrodes were cut into a circular design with a diameter of 10 mm and active materials mass loading controlled in the range of 0.8–1 mg cm^−2^ and a thickness of 13–15 µm (Figure S14, Supporting Information). 1 m NaClO_4_ in EC and DEC (1:1 in volume) with 5% FEC was used as an electrolyte with sodium foil as a counter electrode and the glass fiber (Whatman, GF/D) as a separator. The coin cells were assembled in an argon‐filled glove box (Mikrouna, H_2_O < 0.1 ppm, O_2_ < 0.1 ppm). The galvanostatic charge–discharge test and GITT were carried out using the LANHE CT001 battery test system with the potential range of 0.001–3 V at a constant temperature (25 °C), and the GITT potential profiles were tested for 30 min and relaxation for 2 h. The CV measurements were tested on the Bio‐Logic VSP electrochemical workstation with the potential range of 0.001–3 V and the scanning rate of 0.1 mV s^−1^. The EIS with the frequency ranged from 100 mHz to 100 kHz was performed on the Bio‐Logic VSP electrochemical workstation. The coin‐type sodium‐ion full‐cells were fabricated using LCS‐73 carbon sample as an anode material against NaFe_0.33_Ni_0.33_Mn_0.33_O_2_ (Shanghai Zijian Chemical Technology Co.) as a cathode material in the weight ratio of 1:2.7. The charge–discharge process of the full‐cell was tested at various current rates in the voltage range of 0.5–4 V.

## Conflict of Interest

The authors declare no conflict of interest.

## Supporting information

Supporting Information

## Data Availability

The data that support the findings of this study are available from the corresponding author upon reasonable request.
