# Supplementary material for: Microcrystalline Hybridization Enhanced Coal‐Based Carbon Anode for Advanced Sodium‐Ion Batteries
Source: Adv Sci (Weinh). 2022 May 4;9(20):2200023. doi: 10.1002/advs.202200023 (PMC9284145; doi:10.1002/advs.202200023)
Supplement: Supplementary file 1 — Supporting Information [file ADVS-9-2200023-s001.pdf]

ADVANCED SCIENCE
Open Access

Supporting Information

for *Adv. Sci.*, DOI 10.1002/advs.202200023

Microcrystalline Hybridization Enhanced Coal-Based Carbon Anode for Advanced Sodium-Ion Batteries

*He Chen, Ning Sun, Qizhen Zhu, Razium Ali Soomro and Bin Xu**

Supporting Information

Microcrystalline hybridization enhanced coal-based carbon anode for advanced sodium-ion batteries

*He Chen, Ning Sun, Qizhen Zhu, Razium Ali Soomro, and Bin Xu**

H. Chen, Dr. N. Sun, Prof. Q. Zhu, Dr. R. A. Soomro, Prof. B. Xu*
State Key Laboratory of Organic-Inorganic Composites
Beijing Key Laboratory of Electrochemical Process and Technology for Materials
Beijing University of Chemical Technology
Beijing 100029, China
E-mail: binxumail@163.com, xubin@mail.buct.edu.cn
H. Chen and N. Sun contributed equally to this work.

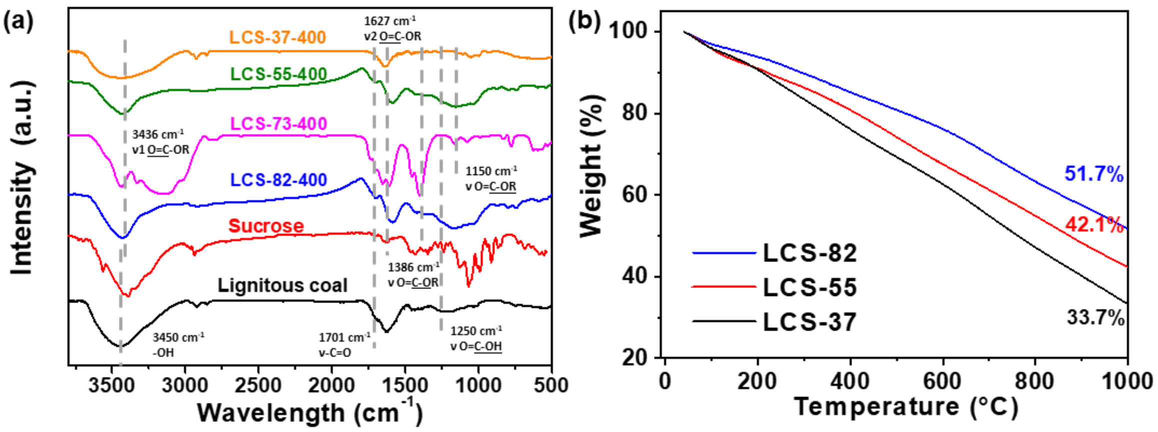

Figure S1. a) Fourier-transform infrared spectroscopy (FTIR) of the precursors: lignite coal, sucrose, and LCS-400. b) TGA curve of the LCS-37, LCS-55, LCS-82.

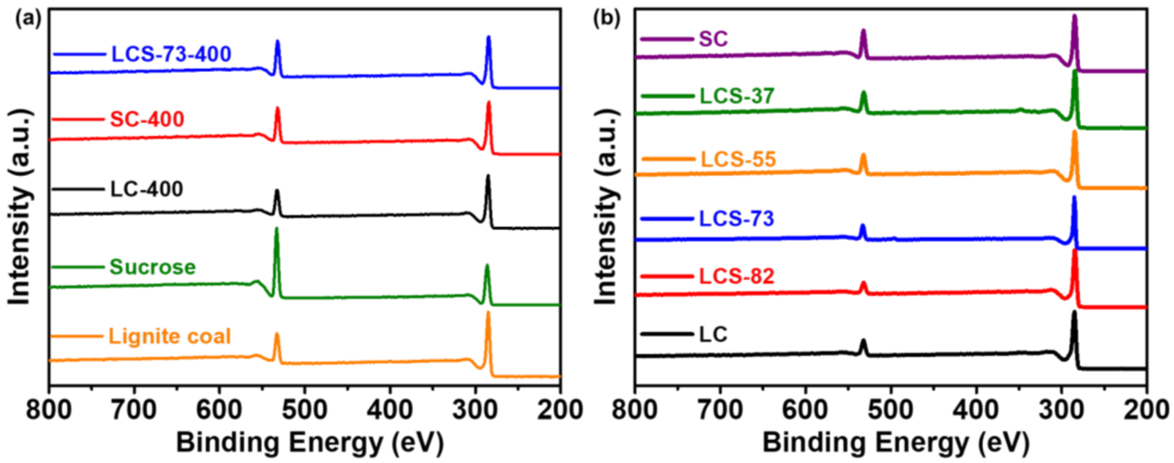

Figure S2. The XPS survey spectra of a) lignite coal, sucrose, LC-400, SC-400, LCS-73-400 and b) carbon materials of LC, LCS-82, LCS-73, LCS-55, LCS-37, SC.

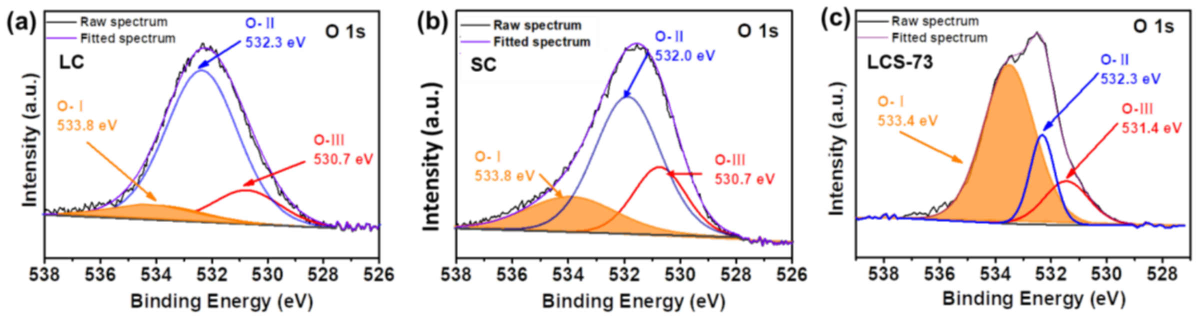

Figure S3. High-resolution O 1s spectrum of a) LC, b) SC and c) LCS-73.

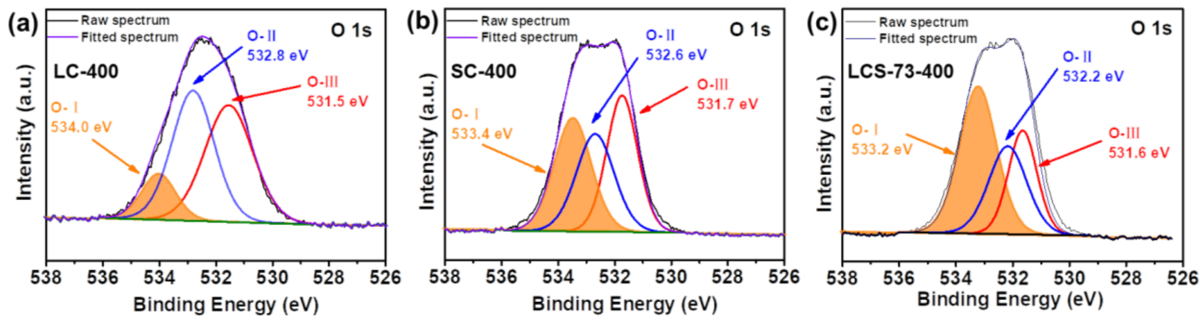

Figure S4. XPS high-resolution O 1s spectrum of a) LC-400, b) SC-400 and c) LCS-73-400.

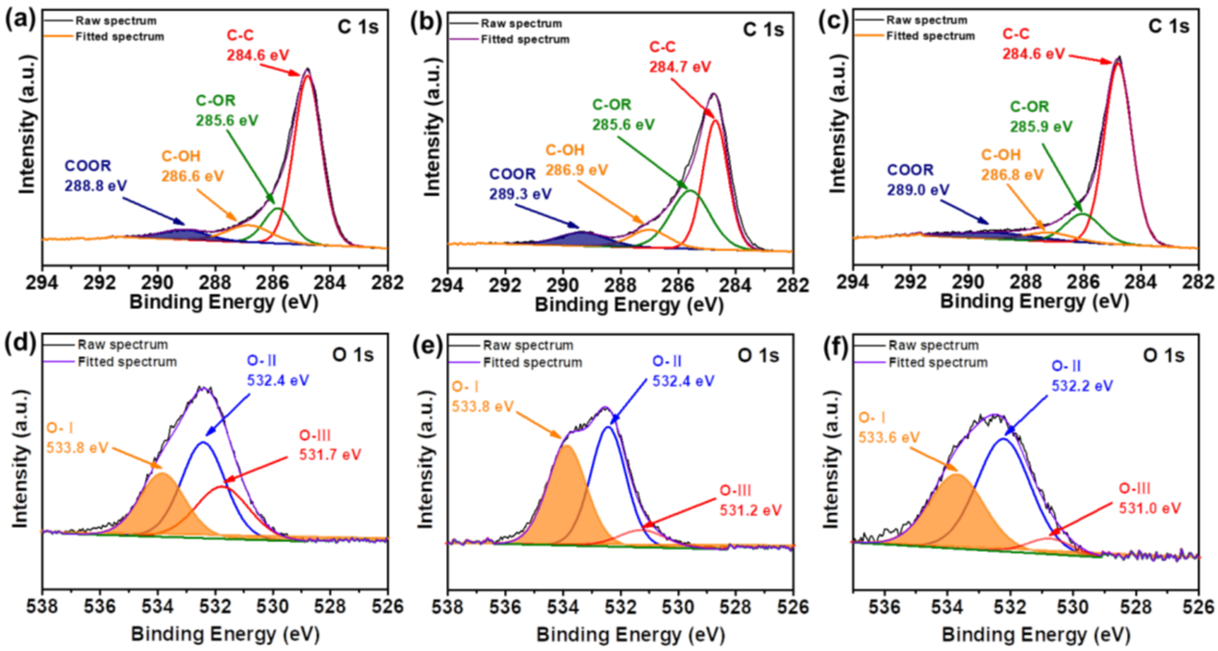

Figure S5. High-resolution C 1s spectrum of a) LCS-37, b) LCS-55 and c) LCS-82. High-resolution O 1s spectrum of d) LCS-37, e) LCS-55 and f) LCS-82.

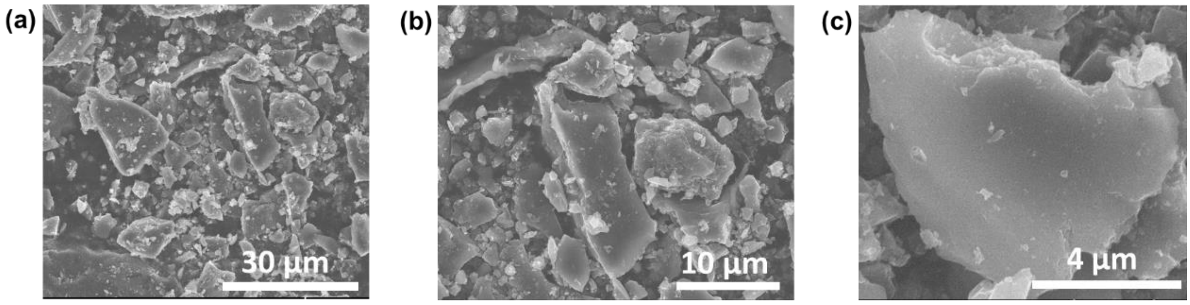

Figure S6. SEM images of LCS-73 carbon particles.

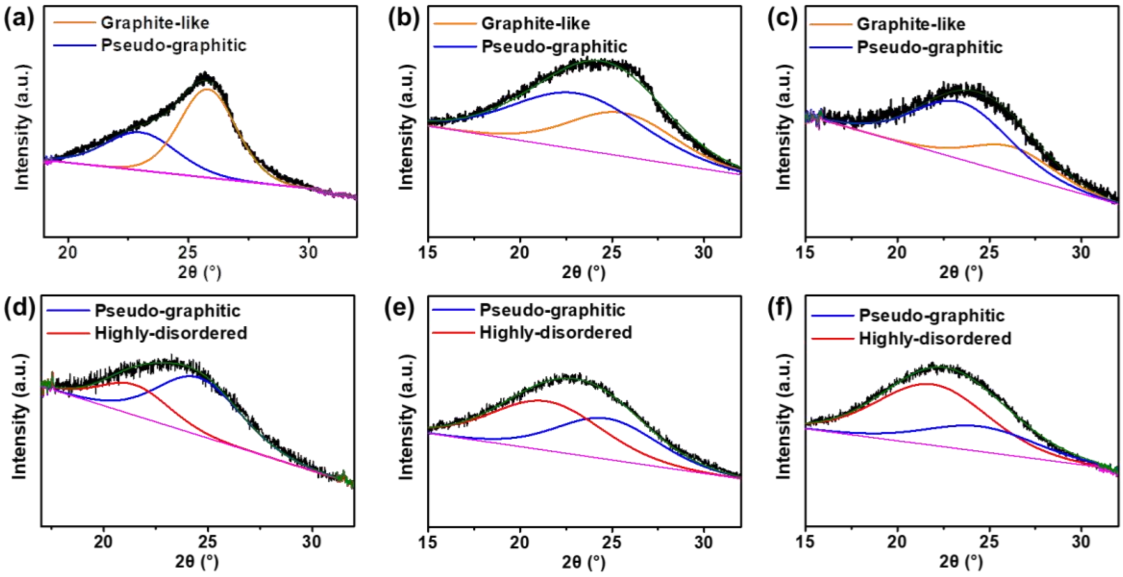

Figure S7. XRD peak fitting for the (002) peaks of a) LC, b) LCS-82, c) LCS-73, d) LCS-55, e) LCS-37, and f) SC.

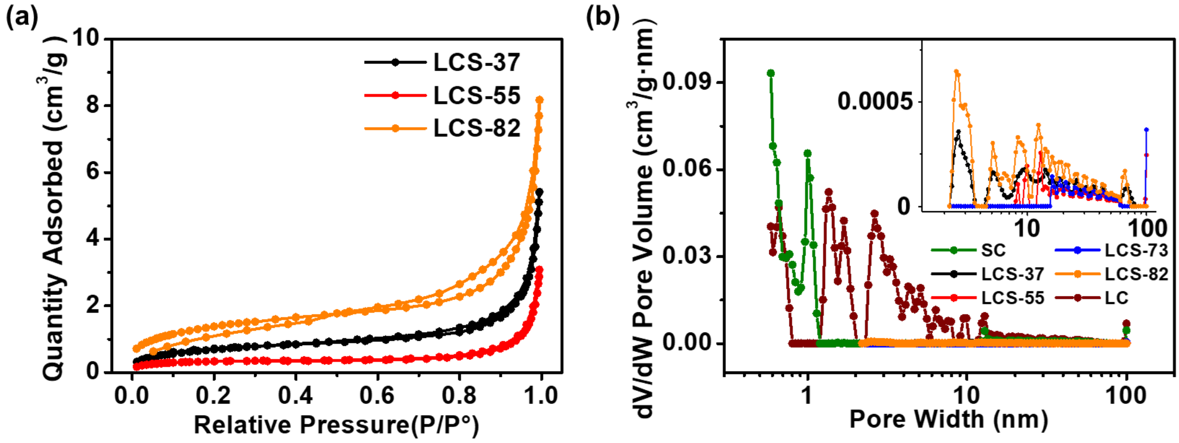

Figure S8. a) Nitrogen adsorption/desorption isotherms of the LCS-37, LCS-55 and LCS-82, and b) pore width distribution of the samples.

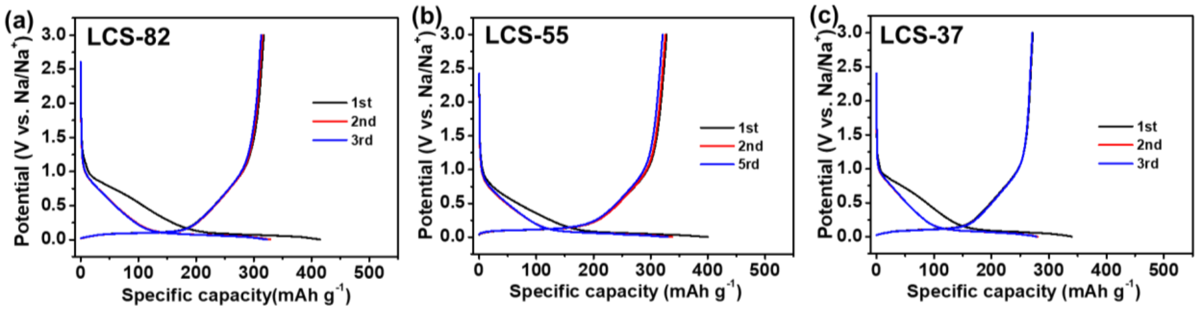

Figure S9. Charge/discharge curves of the samples at 0.03 A g^{-1}: a) LCS-82, b) LCS-55 and c) LCS-37.

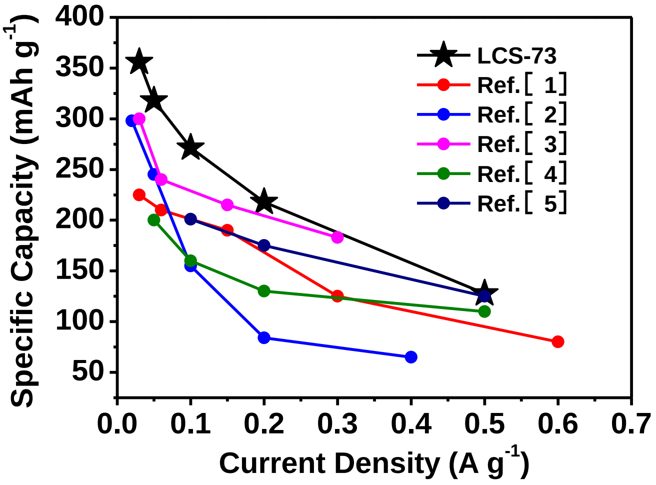

Figure S10. The comparison for electrochemical performance of LCS-73 with previously reported coal-based materials.[1-5]

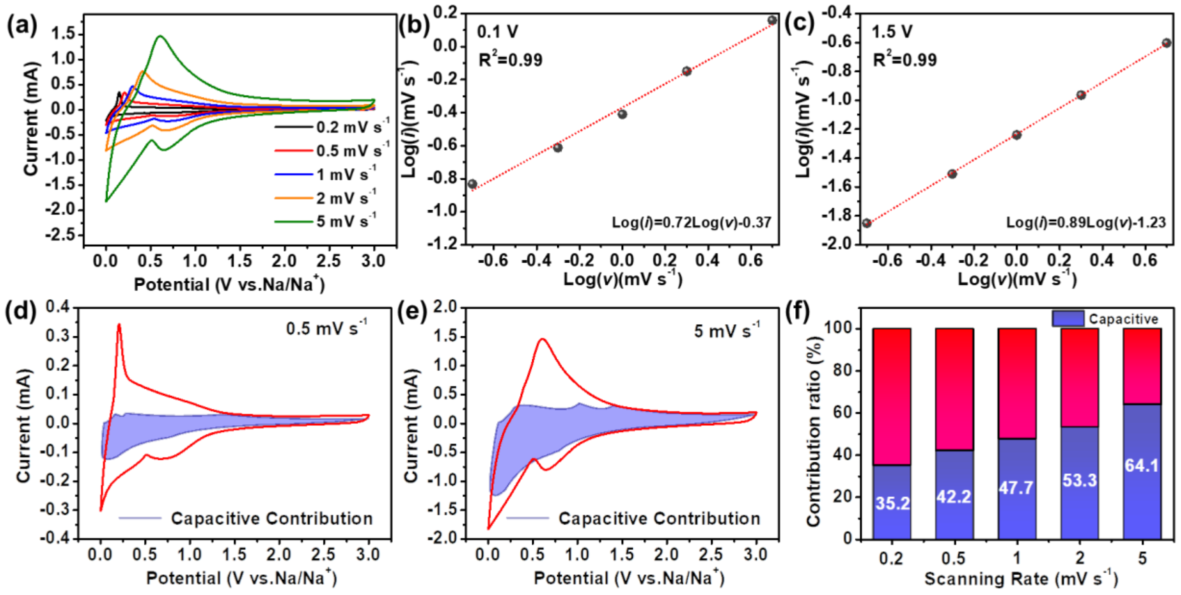

Figure S11. Electrochemical kinetics analysis for LC. a) CV curves at various scanning rates from 0.2 to 5 mV s^{-1}. The correlations of current scan rate (v) and peak current (i) around b) 0.1 V and c) 1.5 V during the discharge process. CV curve with a calculated capacitive contribution at d) 0.5 mV s^{-1} and e) 5 mV s^{-1}. f) Capacitive contributions at various scanning rates.

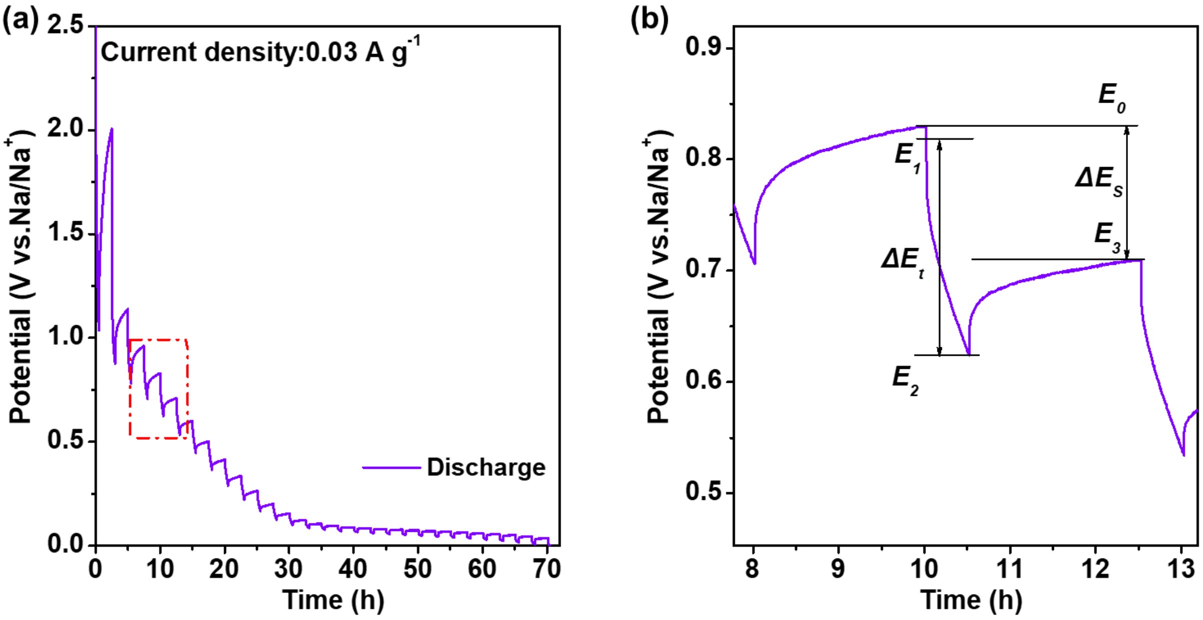

Figure S12. The enlarged GITT graph of LCS-73 in discharge process as the example: ΔE_t is the voltage change of constant current discharging which is calculated by the equal E_1-E_2; ΔE_s is the voltage change caused by the pulse which is calculated by the equal E_0-E_3.

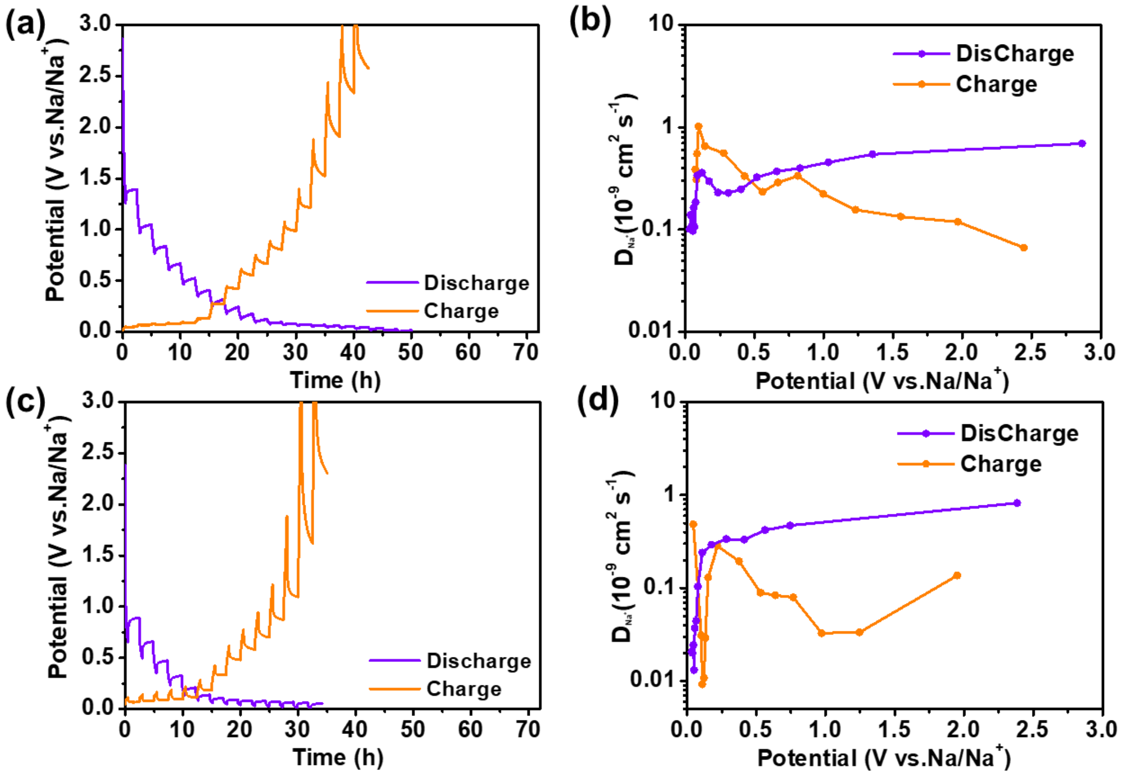

Figure S13. a) GITT potential profiles of LC and b) the corresponding calculated Na^+ diffusion coefficients during the charge-discharge process. c) GITT potential profiles of SC and d) the corresponding calculated Na^+ diffusion coefficients during the charge-discharge process.

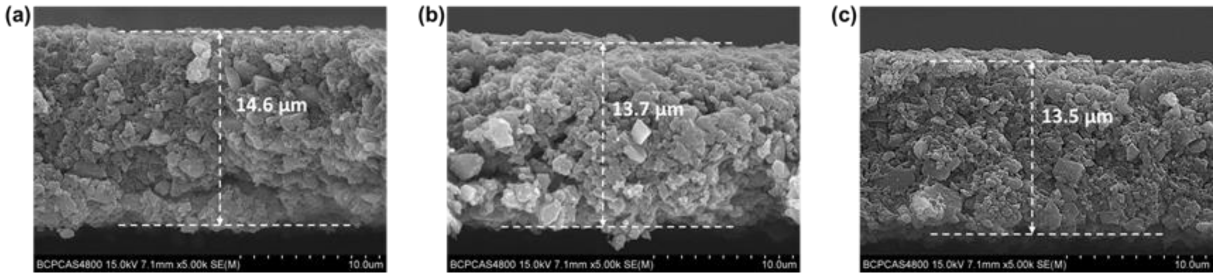

Figure S14. The cross-sectional SEM images of the electrode a) LC, b) SC and c) LCS-73 with the average thickness of 14.6 μm, 13.7 μm and 13.5 μm, respectively.

Table S1. The characterization and electrochemical performance of the carbon samples.

Sample	Theoretical carbonation yield [%][a)]	Actual Carbonation yield [%][b)]	d(002) [nm]	I_G/I_D	Charge Capacity [mAh g^{-1}]	Discharge Capacity [mAh g^{-1}]	ICE [%]	Capacity retention [%]	Capacity at 0.5A g^{-1} [mAh g^{-1}]
SC	-	20.3	0.396	0.495	209	342	61.1	95	30
LCS-37	31.2	32.6	0.388	0.525	272	340	80.0	91	30
LCS-55	38.4	41.8	0.382	0.535	328	400	81.8	91	92
LCS-73	45.7	49.2	0.377	0.541	356	429	82.9	91	114
LCS-82	49.3	50.5	0.361	0.613	317	415	76.4	97	139
LC	-	56.6	0.345	0.628	290	484	59.9	96	88

[a)] The theoretical carbonation is calculated based on actual carbonation yield of SC and LC;
[b)] The actual carbonation yield is calculated based on the mass ratio after and before carbonization at 1200 ° C.

Table S2. Fitting peak area proportion of C 1s and O 1s spectra in XPS pattern of the carbon samples.

Samples	C 1s [%]				O 1s [%]		
	C-C	C-OR	C-OH	C-OOR	O-I (-COOR)	O-II(-OH)	O-III(C=O)
LC-400	47.64	32.15	11.28	8.93	12.36	43.14	44.50
S-400	58.12	22.87	8.86	10.15	34.75	30.49	34.76
LCS-73-400	69.60	11.90	7.04	11.46	46.56	28.29	25.12
LC	67.17	13.98	13.56	5.29	8.58	75.80	15.62
SC	71.60	13.60	8.79	6.01	20.16	57.13	22.71
LCS-82	71.85	14.71	6.05	7.39	37.85	56.00	6.15
LCS-73	52.37	19.54	18.44	9.65	66.39	17.81	15.80
LCS-55	55.02	33.26	10.07	9.47	43.94	47.32	8.74
LCS-37	67.80	14.71	10.93	6.56	29.34	42.64	28.1

Table S3. The elemental analysis results of the precursors and the carbon materials.

Samples	C [wt%]	H [wt%]	O [wt%]	N [wt%]
Lignite coal	73.24	4.53	21.44	0.79
Sucrose	42.20	6.59	51.21	0
LC	93.01	0.45	5.92	0.62
SC	87.94	0.20	12.06	0
LCS-37	92.45	0.34	6.41	0.76
LCS-55	93.02	0.32	6.04	0.80
LCS-73	93.93	0.25	4.96	0.86
LCS-82	94.80	0.20	4.10	0.90

Table S4. The BET surface area and pore volumes of the carbon samples.

Sample	BET surface area [m^2 g^{-1}]	Pore volumes [cm^3 g^{-1}]
SC	80.69	0.1103
LCS-37	2.49	0.0084
LCS-55	1.09	0.0048
LCS-73	1.48	0.0053
LCS-82	4.93	0.0126
LC	169.52	0.2367

Table S5. Equivalent circuit fitting calculation results of electrochemical impedance spectroscopy.

Sample	R_{Ω} [Ω]	R_{ct} [Ω]
SC	8.4	181.0
LCS-37	4.5	160.4
LCS-55	5.4	140.3
LCS-73	5.5	107.4
LCS-82	8.1	94.1
LC	7.9	91.6

References

[1] Y. Li, Y.-S. Hu, X. Qi, X. Rong, H. Li, X. Huang, L. Chen, *Energy Storage Mater.* **2016**, 5, 191.

[2] H. Lu, S. Sun, L. Xiao, J. Qian, X. Ai, H. Yang, A.-H. Lu, Y. Cao, *ACS Appl. Energy Mater.* **2019**, 2, 729.

[3] M. Kang, H. Zhao, J. Ye, W. Song, H. Shen, J. Mi, Z. Li, *J. Mater. Chem. A* **2019**, 7, 7565.

[4] Z. Zhuang, Y. Cui, H. Zhu, Y. Shi, Q. Zhuang, *J. Electrochem. Soc.* **2018**, 165, A2225.

[5] R. Liu, Y. Li, C. Wang, N. Xiao, L. He, H. Guo, P. Wan, Y. Zhou, J. Qiu, *Fuel Process. Technol.* **2018**, 178, 35.
